# wrmXpress: A modular package for high-throughput image analysis of parasitic and free-living worms

**DOI:** 10.1371/journal.pntd.0010937

**Published:** 2022-11-18

**Authors:** Nicolas J. Wheeler, Kendra J. Gallo, Elena J. G. Rehborg, Kaetlyn T. Ryan, John D. Chan, Mostafa Zamanian

**Affiliations:** 1 Department of Pathobiological Sciences, University of Wisconsin-Madison, Madison, Wisconsin United States of America; 2 Department of Chemistry, University of Wisconsin-Oshkosh, Oshkosh, Wisconsin United States of America; University of Glasgow, UNITED KINGDOM

## Abstract

Advances in high-throughput and high-content imaging technologies require concomitant development of analytical software capable of handling large datasets and generating relevant phenotypic measurements. Several tools have been developed to analyze drug response phenotypes in parasitic and free-living worms, but these are siloed and often limited to specific instrumentation, worm species, and single phenotypes. No unified tool exists to analyze diverse high-content phenotypic imaging data of worms and provide a platform for future extensibility. We have developed wrmXpress, a unified framework for analyzing a variety of phenotypes matched to high-content experimental assays of free-living and parasitic nematodes and flatworms. We demonstrate its utility for analyzing a suite of phenotypes, including motility, development/size, fecundity, and feeding, and establish the package as a platform upon which to build future custom phenotypic modules. We show that wrmXpress can serve as an analytical workhorse for anthelmintic screening efforts across schistosomes, filarial nematodes, and free-living model nematodes and holds promise for enabling collaboration among investigators with diverse interests.

## Author summary

Advances in automated microscopy have led to the proliferation of high-throughput experimental techniques to screen drugs for activity against parasitic worms, which infect billions of people around the world. Most of the software available to analyze high-content microscopy datasets is optimized for cell lines and is rarely applicable for images of parasitic worms. Applications that have been designed with worms in mind often focus on single phenotypes, such as worm motility or mortality, and are not built for extension to other phenotypic outputs. We have developed wrmXpress, a software package that provides a unified framework for analyzing a wide variety of worm phenotypes, including neuromuscular activity, fecundity, mortality, development/size, and feeding. wrmXpress can analyze images and videos produced by multiple imaging platforms and is open source, written in popular interpreted computer languages with extension in mind, providing a foundation for collaborative efforts that produce novel phenotypes and require bespoke software development.

## Introduction

The past decade has seen the development of a variety of software for the acquisition and analysis of high-throughput and high-content imaging data of roundworms and flatworms, both free-living and parasitic [1,2]. New instrumentation and analytical capabilities have laid the foundation for a new era of phenotype-driven screening for anthelmintic compounds.

Early iterations of image-based screening focused on gross worm movement, using a number of different approaches to quantify motility, including sparse measures of optical flow and frame-by-frame pixel variation [3–7]. Optical flow was found to be robust to a number of diverse nematode and flatworm parasites and has been the basis for some of the largest phenotypic screening efforts to-date [8–10]. Other developments in high-content imaging, sometimes combined with the employment of fluorescent stains to reveal fine-scale phenotypes, now allow for the quantification of detailed morphological and molecular features that can be used for image-based classification strategies [11–14]. Open source packages have been developed to more readily handle large imaging datasets and provide quick readouts for quality control of entire experiments, plates, wells, and even individual worms [15].

Not unexpectedly, individual labs often develop their pipelines to suit their own needs. These pipelines tend to focus on specific species and stages, require specific instrumentation, and demand an advanced grasp of compiled languages, resulting in siloed development and redundant rather than collaborative engineering efforts. There have been recent developments that unify parts of these efforts for the capture of phenotypes in model nematodes [15]. No package has yet to bring multiple phenotypes (i.e., motility and morphology) into a single framework that prioritizes flexibility across free-living and parasitic worms. Here, we present wrmXpress, a modular open source package that consolidates multiple analytical approaches. It is written entirely in popular, open source, interpreted programming languages (Python and R) and is configured with a human-readable markup language (YAML). It is containerized for deployment across a wide variety of compute platforms (both distributed and isolated), enabling collaboration and reproducibility. Finally, while it ships with a range of phenotype pipelines, it establishes a foundation for extension to additional analyses and species, including future image-based deep learning applications.

## Methods

### Ethics statement

Animal work was carried out with the oversight and approval of UW-Madison Research Animal Resources and Compliance (RARC), adhering to the humane standards for the health and welfare of animals used for biomedical purposes defined by the Animal Welfare Act and the Health Research Extension Act. Experiments were approved by the UW-Madison School of Veterinary Medicine IACUC committee (approved protocol #V006353-A08).

### Protocol and data availability

Most experimental protocols for generating the sample imaging data presented here can be found in separate manuscripts. Multivariate phenotyping of *Brugia* spp. microfilariae and adults (including motility, viability, and fecundity) and *Caenorhabditis elegans* feeding and development assays are described elsewhere [16,17]. Schistosoma *mansoni* motility and fecundity assays are described below.

wrmXpress v1.3.0 is publicly available at https://github.com/zamanianlab/wrmXpress, which includes a Conda environment file to install dependencies. A Docker image that includes all dependencies is publicly available at https://hub.docker.com/r/zamanianlab/chtc-wrmxpress. Example imaging data for each module is available as a Zenodo repository (10.5281/zenodo.7116648).

### Worm strains and sources

*Brugia malayi* and *Brugia pahangi* life cycle stages were obtained through the NIH/NIAID Filariasis Research Reagent Resource Center (FR3); morphological voucher specimens are stored at the Harold W. Manter Museum at University of Nebraska, accession numbers P2021-2032 [18]. Nematode parasites were maintained at 37°C with 5% atmospheric CO_2_ in RPMI 1640 culture media (Sigma-Aldrich, St. Louis, MO) with penicillin/streptomycin (0.1 mg/mL, Gibco, Gaithersburg, MD) and FBS (10% v/v, Gibco) unless otherwise stated.

Female Swiss Webster mice infected with *S*. *mansoni* NMRI cercariae were euthanized with CO_2_ at 7 weeks post-infection, and adult worm pairs were retrieved from the mesenteric vasculature. Harvested worms were washed and maintained in high-glucose DMEM (ThermoFisher, Waltham, MA) supplemented with HEPES (25 mM), 5% heat-inactivated FCS (Sigma-Aldrich) and pen/strep (100 units/mL).

*C*. *elegans* were maintained using established rearing protocols. Strains N2, *unc-122p*::*GFP*, and ZAM11 (*gar-3(gk305) V*, *maz11Ex[myo-2p*::*Bma-gar-3*::*unc-54 3’UTR; myo-2p*::*GFP]*) were used for all example images [17]. Transgenic strains were generated using standard microinjection protocols with plasmid DNA [19].

### *Schistosoma mansoni* motility and fecundity assays

Adult *S*. *mansoni* pairs (fecundity) or individual males/females (motility) were manually sorted into a 96-well plate (Greiner Bio-One, Frickenhausen, Germany) with culture media, which were then top-filled. For fecundity experiments, worm pairs were treated with 0.01% DMSO or varying concentrations of praziquantel (Santa Cruz Biotechnology, Dallas, TX, with a final concentration of 0.01% DMSO) and were incubated at 37°C with 5% atmospheric CO_2_ for 72 hours.

### Image acquisition and analysis

Images were acquired with an ImageXpress Nano (Molecular Devices) or a bespoke imaging platform previously described [10]. *C*. *elegans* were imaged at 2x with transmitted light and GFP/TxRed where applicable. For *B*. *malayi* microfilariae motility, 10 frames (~3.3 frames per second) were acquired at 4x with transmitted light with the field of view focused on the center of the well; for *B*. *malayi* microfilariae viability, wells were imaged at 4x with with a GFP filter set, tiled 2x2 to acquire the entire well. For *B*. *pahangi* fecundity, progeny was imaged at 4x with transmitted light, tiled 2x2. For *S*. *mansoni* adult motility, wells were acquired for 60 frames at 2x with transmitted light and 2x binning. For *S*. *mansoni* fecundity, plates were first imaged at 2x with transmitted light with worm pairs remaining in the wells, after which worms were manually removed and imaging was repeated.

Raw images were exported with MetaXpress v6 and stored on the UW-Madison Research Drive in an uncompressed state. When analyzed using a distributed computing system, images were transferred to the UW-Madison Center for High-Throughput Computing (CHTC) submit servers. Jobs were submitted and managed with HTCondor [20]. HTCondor submit scripts are publicly available at https://github.com/zamanianlab/chtc-submit/tree/main/imgproc.

### Building a deep learning segmentation model for *C*. *elegans* using Cellpose

Cellpose is a deep learning algorithm used for the segmentation of objects in microscopy images [21]. To our knowledge, a Cellpose model has not been trained for segmenting *C*. *elegans* of varying size, shape, and stage. We selected 139 training images from a range of previous experiments [16,17] to include a diverse population of worms, varying in size and shape (straightened or curved with paralytics), and used the Cellpose 2.1.0 GUI to manually segment every worm (>4000 objects). Training images were categorized by the paralytic that was used on the worms (sodium azide or 1P2P) and the sizes of the worms in the image (large, small, or mixed). Cellpose models were trained from scratch, with a learning rate of 0.2 and weight decay of 0.000001 with 500 epochs. An individual model was trained for each image category in addition to a global model that incorporated all 139 images. Two to four images from each category were manually segmented and retained as hold-out images for model evaluation (S1 Fig).

### Building a machine-learning classifier for *C*. *elegans*

Worm images straightened by the Worm Toolbox in CellProfiler from six different experiments were manually classified as Single worm, Partial worm, Multiple worms, or Debris (8535 objects from 201 images in total). Each object was associated with 25 area/shape features and 15 intensity features output by CellProfiler v4.2.1, and the features and training data were used to build several classification models using a variety of approaches, including the boosted frameworks XGBoost [22,23] and LightGBM [24]. These were implemented using the tidymodels package in the R statistical software [25]. Highly correlated predictors and predictors with near-zero variance were removed using tidymodels functions. Care was taken to balance the dataset by worm classification and the size of single worms, and lowly represented classes were upsampled with the SMOTE method [26]. Data was split into training/testing sets, and the training results were evaluated using ten-fold cross validation. Model hyperparameters were tuned using grid searches. Tuned models were fit to the cross-fold testing set and evaluated using the area under the receiver-operator curve (ROC AUC). Complete modeling code is available in the wrmXpress package so that users can generate their own classifiers.

## Results

### wrmXpress is user-friendly, modular, and extensible

wrmXpress is a unified framework for analyzing worm imaging data. It comes packaged with a variety of phenotyping modules matched to specific experimental setups, including motility and viability for parasites and *C*. *elegans*, feeding rate and development in *C*. *elegans*, and fecundity for parasites. The package is extensible using open source Python libraries or new CellProfiler pipelines.

**Fig 1:**
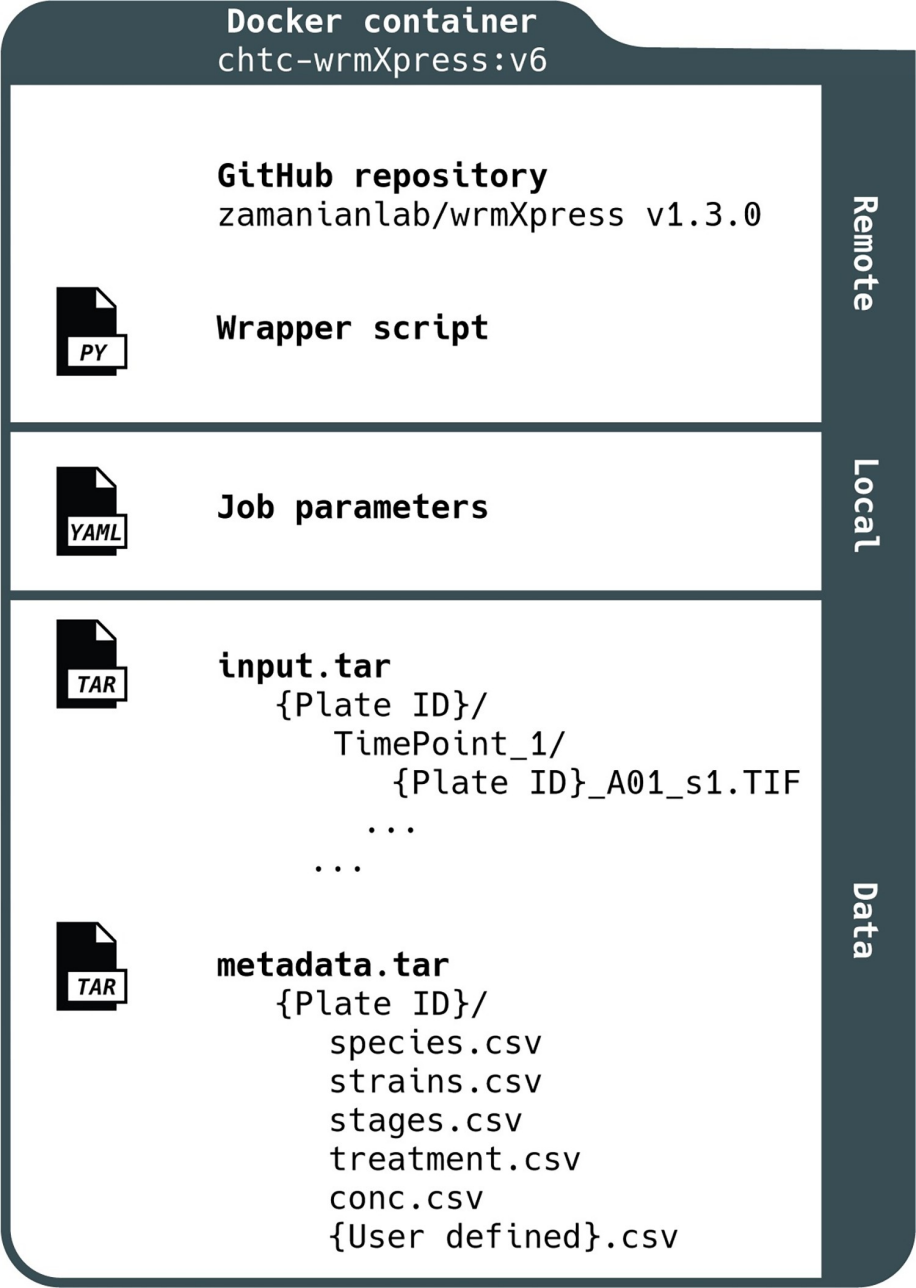
Schematic of wrmXpress. wrmXpress consists of code that is held in a public GitHub repository (including the master wrapper script), job parameters that are edited locally, and external data and metadata. The structure of input data and metadata requires specific formats in order for wrmXpress to complete without error.

**Fig 2:**
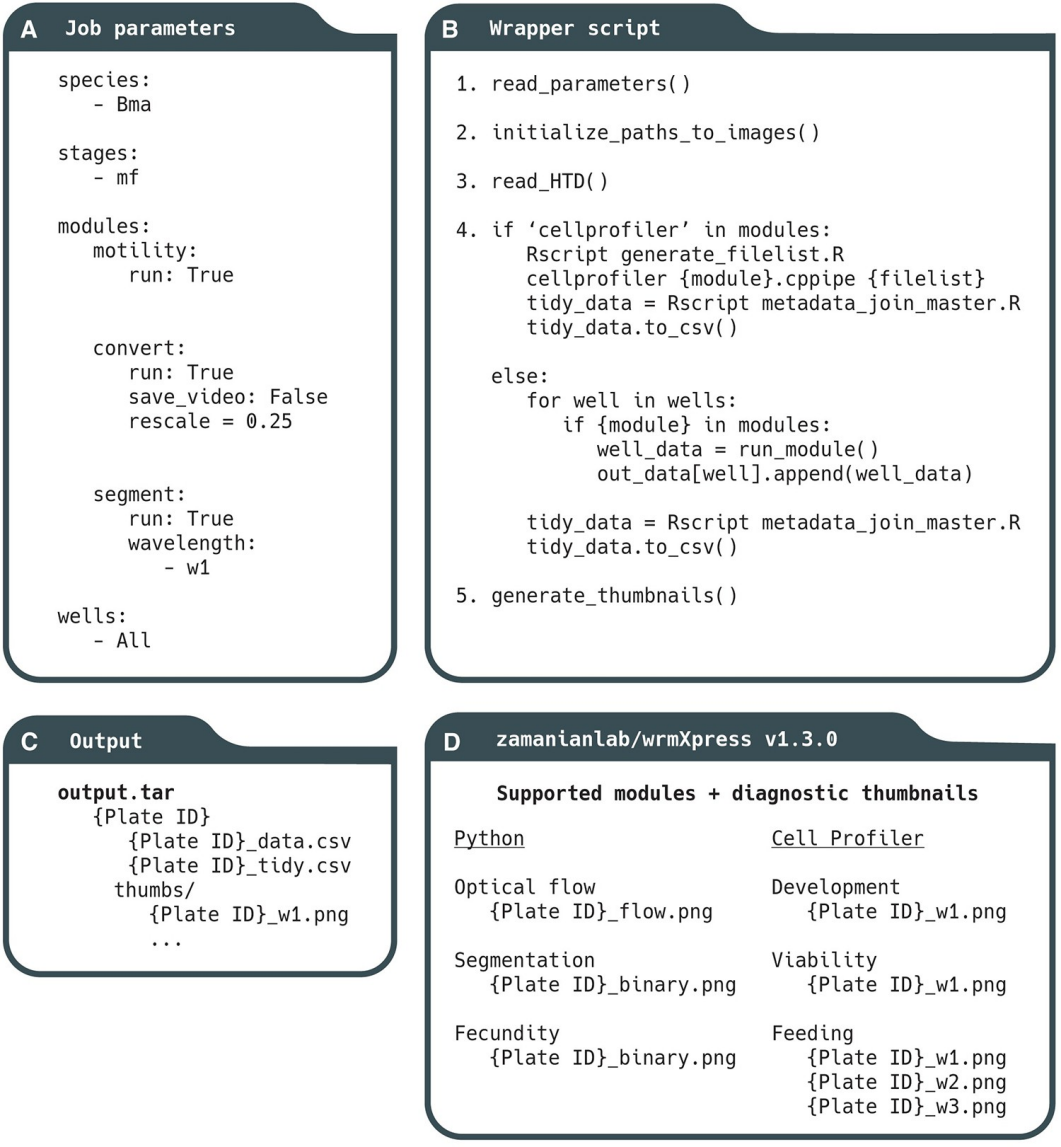
Constituents of the wrmXpress workflow. (A) Jobs are parameterized with a user-generated YAML file, which includes species and stage information, and allows for the selection of Python or CellProfiler modules. (B) The wrapper script controls the implementation of wrmXpress. (C) Output data includes raw data, raw data with joined metadata, and diagnostic thumbnail images. (D) wrmXpress comes packaged with 6 distinct analytical modules.

The package combines code, user-generated job parameters, and input data/metadata (Fig 1), each of which is passed to a Docker container that runs the pipeline. It is implemented with a single command (e.g., python wrmXpress/wrapper.py params.yml {plate_dir}), and modules are configured and initialized with the YAML parameters file that designates the worm species and stage, run-time options, and the modules to be run (Fig 2A). A user can choose to analyze an entire plate or a selected subset of wells. The wrapper.py script integrates these selections and runs the proper modules and commands (Fig 2B). Output data is written to a directory with raw output, a tidied output that joins well-based experimental metadata, and thumbnail images to assist with quality control and error diagnosis (Fig 2C).

### wrmXpress usage

wrmXpress is designed such that each module outputs a single phenotypic value per well, or multiple values per well or object if using a CellProfiler pipeline. For instance, for a motility experiment that could use worm area as a normalization coefficient, both the motility and segmentation modules can be selected, which will independently calculate the raw optical flow and total worm area per well. Each value is then concatenated to a final output file that includes metadata and per-module measurements.

At the start of a wrmXpress run, the user-generated parameters provided by the YAML are read and organized (Fig 2B, step 1). Paths to relevant image and metadata files are populated, and modules are selected (Fig 2B, step 2). It is during this stage that wells of interest can be selected in order to reduce runtime in case of contamination, empty wells, or during testing. Since not all modules are compatible (for instance, some require multiple time points and some require multiple wavelengths), some light checking of parameters and input data is performed in order to avoid module clashes and to ensure a correct pairing between modules and input data. Finally, the plate’s HTD file, a machine-generated configuration file that reports imager settings, is parsed (Fig 2B, step 3). These imager configurations are used in some downstream modules, like stitching of tiled images. If an HTD file is unavailable, wrmXpress will attempt to generate one with the metadata available in the input.

Once paths and parameters are organized, the wrapper script loops through the selected wells and iteratively calls the functions for each selected module (Fig 2B, step 4). For CellProfiler pipelines, an R script utilizes the populated file paths to automatically generate the CSV that is used by CellProfiler’s LoadData module. CellProfiler is then called in headless mode. Each pipeline must also include the ExportToSpreadsheet module, which collects the well and/or object-based data and writes it to a CSV. Finally, another R script joins user-provided experimental metadata to the output CSV to create a final tidy data file.

For bespoke Python modules, less preparation is required. As the wrapper iterates through wells, each module is called independently of other modules. After completion of a well, the module will return a single phenotypic value, which is added to a dictionary of values that is dynamically updated. After iterating through all selected wells, the dictionary is written to a CSV, and the data is tidied as above.

After CellProfiler pipelines or Python modules are finished, diagnostic thumbnails are generated and formatted in an array matching the shape of the input plate. By default, a thumbnail will be created for each included wavelength, and specific modules generate relevant diagnostics to help evaluate module performance.

### Analytical modules for worm motility, area, development, viability, fecundity, and feeding

**Fig 3:**
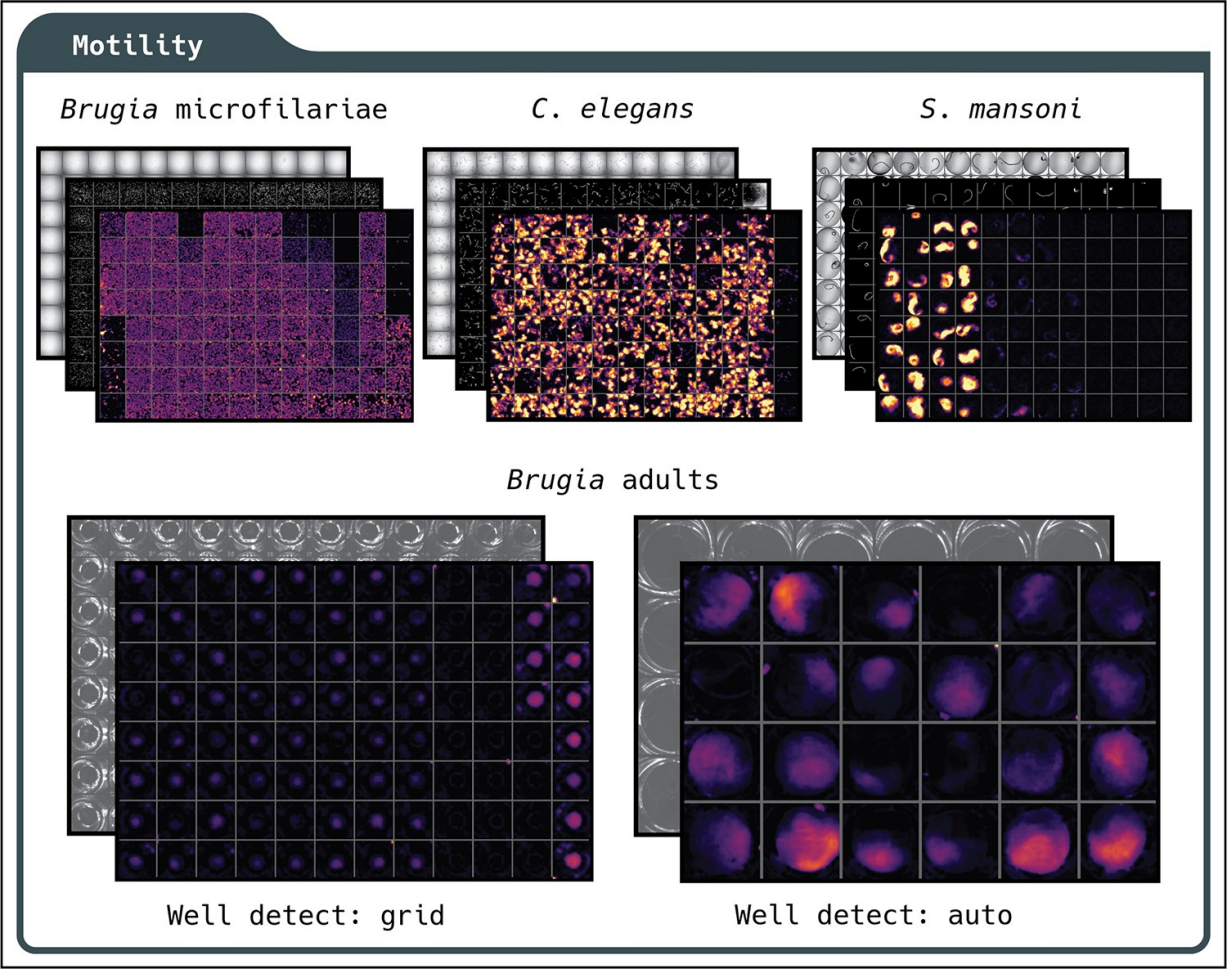
Using wrmXpress to measure motility of diverse worms imaged using a variety of parameters and approaches. Motility of *B*. *malayi* microfilariae, *C*. *elegans* adults, *S*. *mansoni* adults, and *Brugia* spp. adults. Top images represent data acquired with a high-content imager, and bottom images represent data acquired with a simple camera imaging an entire plate on a glass stage. For whole-plate imaging, wells can be split automatically using well identification or using a pre-established grid based on user-input values. Diagnostic images include a single frame of transmitted light, binary segmented worms, and a flow cloud.

wrmXpress comes packaged with six individual modules that enable a wide range of out-of-the-box functionalities (Fig 2D). Motility measurements are implemented using a dense measure of optical flow (Farneback’s method [27]). Dense flow for worm motility has been used elsewhere [10], and offers a richer output than previous optical flow-based implementations that prioritized a sparse feature set (the Lucas-Kanade method [28]). Focusing on a sparse feature set enabled real-time tracking of worms [3,4], but given that real-time tracking is not a priority in high-throughput settings, wrmXpress opts for the more data-rich option. A unitless measure of motility is calculated by summing the magnitude of the flow vectors across *n*-1 frames (where n is the total number of frames in the video), and then summing the sum across all pixels. Thus, flow is a function of video length as well as each frame’s height and width. This algorithm has been tested for *Brugia* spp. microfilariae and adults, *C*. *elegans* L1s and adults, and *S*. *mansoni* adult males and females (Fig 3). A “flow cloud” diagnostic thumbnail is created for each well, providing an image representation of the motility over the entire course of the video. The flow cloud is representative of true motility values for all worms tested (S2 Fig). This diagnostic can also alert investigators to plate effects or heterogeneity between wells.

Motility measurements on wells with multiple worms can be normalized by dividing the motility value by the worm area, which is calculated by the segmentation module. We have found that a simple algorithm incorporating Sobel edge detection, Gaussian blur, and Otsu’s thresholding method performs well for a variety of vermiform objects (including all nematodes so far tested) [29,30]. For larger worms that are less optically translucent (e.g., *S*. *mansoni* adults), we implement Gaussian blur followed by a simple percentile threshold. The percentile and σ for the Gaussian kernel may need to be adjusted in accordance with varying illumination parameters, but the defaults have been robust in our hands (1.5% and σ = 1.5). For *S*. *mansoni* adult females or male/female pairs, which eject a variety of debris in culture, an object size filter has been implemented. The final binary segmented image is also written out as a diagnostic thumbnail (Figs 3 and S2).

Most *in vitro* phenotyping of *Brugia* spp. adults occurs in 24-well plates, which can be recorded with the entire plate in the field of view. We implemented a well-finding algorithm that automatically crops each well and measures its optical flow, similar to the WormAssay approach [3]. For plate formats with >24 wells that are difficult to distinguish, we crop using a simple grid based on the known number of rows and columns. This approach may be preferred for larger adult parasites that can be imaged with little or no magnification with the entire plate in view, saving disk space and computation time.

**Fig 4:**
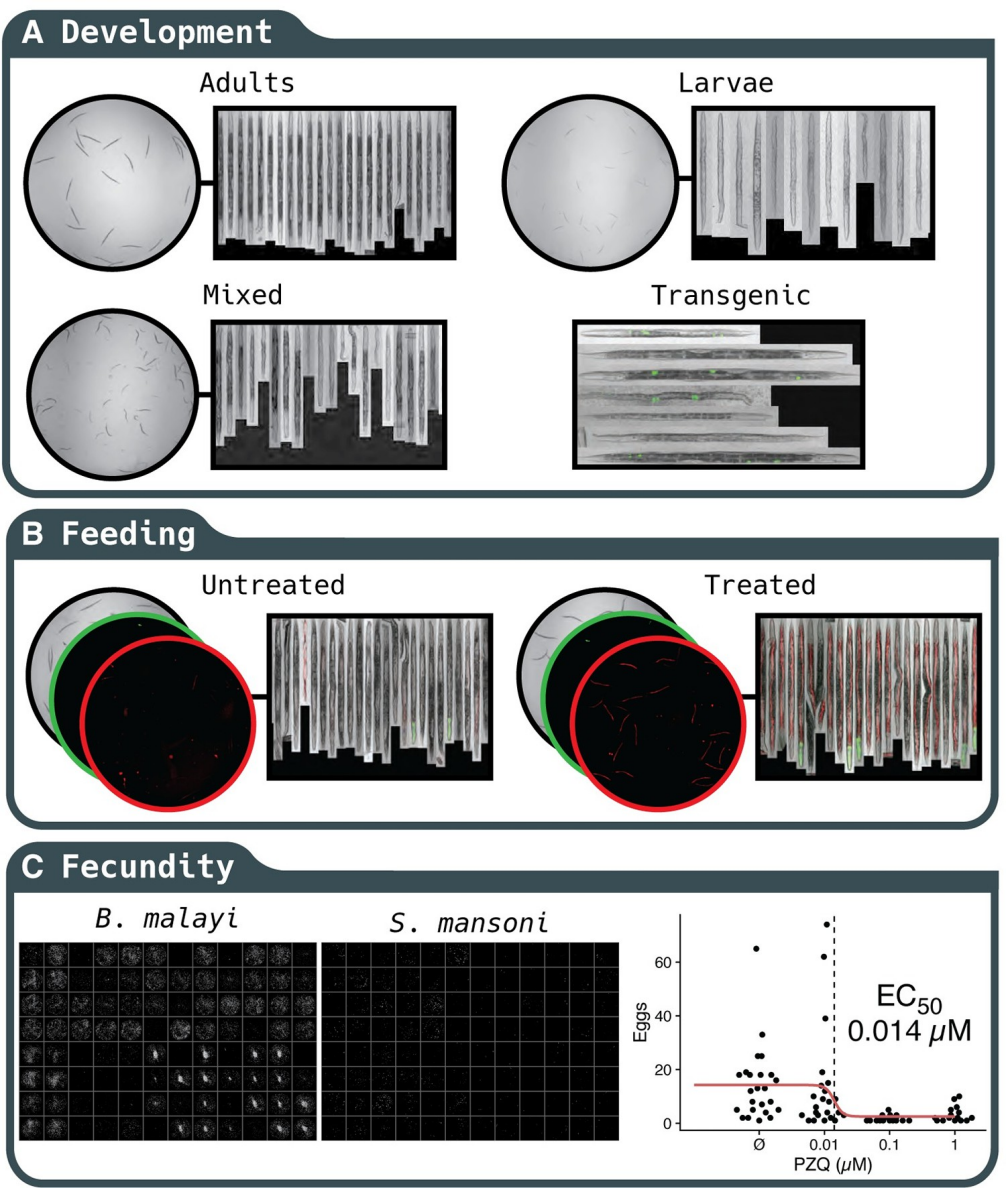
Examples of phenotypes that can be analyzed with wrmXpress. (A) An analytical module is available for measuring *C*. *elegans* development, which performs well with mixed populations. Classification of transgenic worms (*unc-122p*::*GFP*) is also implemented. (B) Quantification of *C*. *elegans* feeding using fluorescent dyes, which can be measured in the worm intestine. (C) Quantification of the *in vitro* fecundity of *Brugia* spp. and *S*. *mansoni* adults.

**Fig 5:**
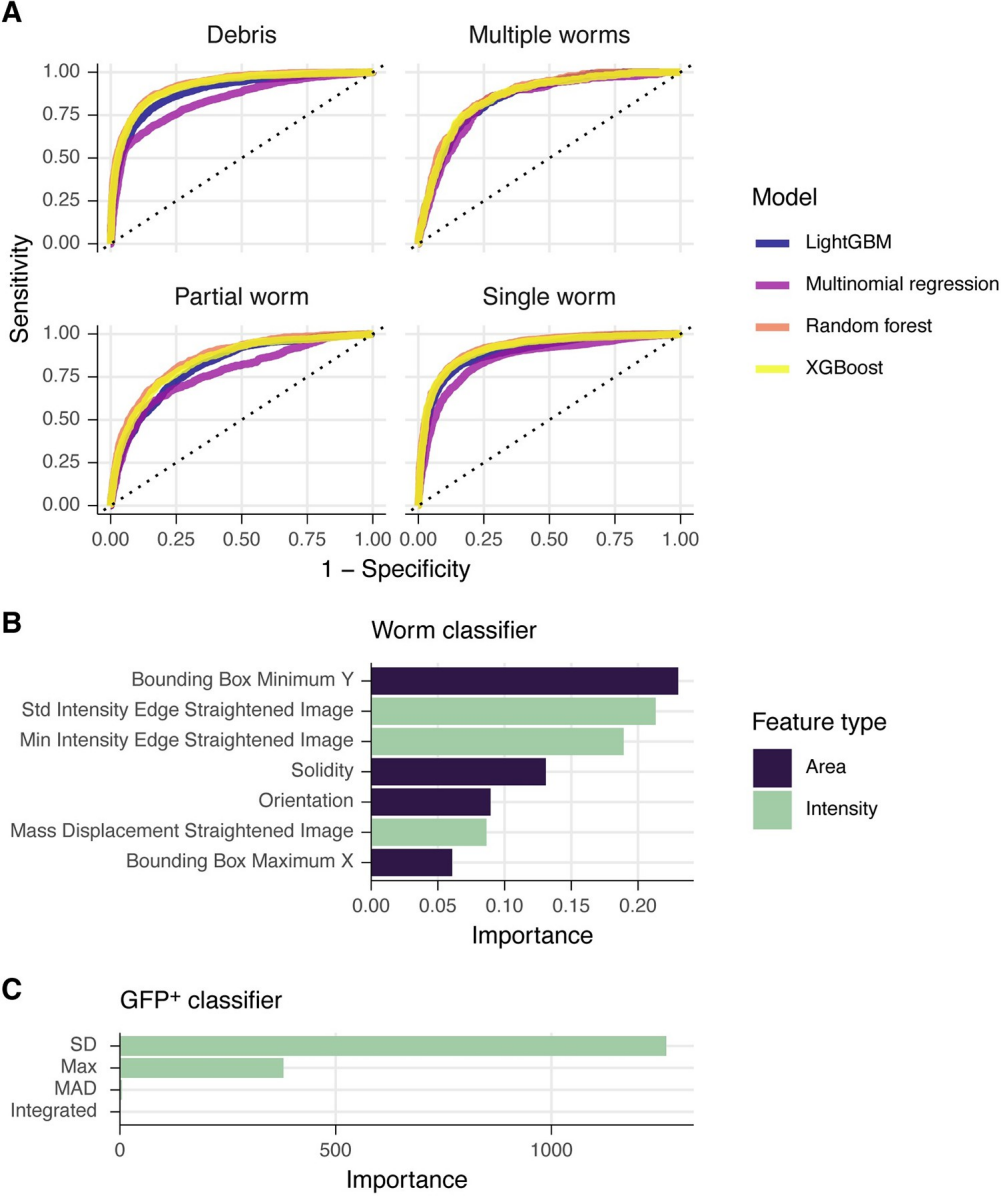
Statistical models used to classify and filter straightened *C*. *elegans*. (A) Evaluation of models classifying segmented and straightened “worms” as debris, multiple worms, a partial worm, or a single worm. (B) Variable importance plot for the tuned XGBoost model from (A). Green bars indicate intensity features, purple indicate area/shape features. (C) Variable importance plot for a tuned random forest to classify GFP+/- worms (*unc-122p*::*GFP*).

Integration of CellProfiler pipelines further extends the capabilities of wrmXpress, which comes with pre-built pipelines for analyzing *C*. *elegans* development and feeding, each of which can be used on mixed populations of transgenic worms with a fluorescent marker (Fig 4A and 4B). These pipelines take advantage of the WormToolbox plugin, which incorporates user-generated worm models to untangle worms [11]. For the wrmXpress development module, a number of innovations were necessary to prepare the pipeline for identifying worms that greatly varied in size and shape, as drug treatment of synchronized worms can lead to mixed populations of worms in a single well (Fig 4A). First, we leveraged the deep learning framework of Cellpose to segment *C*. *elegans* [21]. Second, we trained a post-processing classification model that used object shape and intensity features to classify untangled worms as a single worm, partial worm, multiple worms, or debris. We tuned, trained, and evaluated a variety of machine learning and statistical models and selected a gradient boosted tree due to its performance and speed of classification on experimental data (Fig 5A). The trained model used the minimum y (length) of the bounding box, the minimum and standard deviation of the intensity of the edge pixels, and the solidity (the ratio of the contour area to its convex hull area), among others, as the most important variables (Fig 5B). The model achieved an area under the receiver-operator characteristic curve (AUC ROC) of 0.827 and a sensitivity of 0.609 (S1 File). When fit to annotated holdout data and retaining only those predicted to be single worms, the model removed 93% of the debris, 89% of multiple worms, 93% of partial worms, and only 18% of single worms, substantially enriching for objects of interest. In our hands, using a single, less stringent worm model in CellProfiler followed by post-processing filtration decreased runtime and ensured that smaller worms were captured. All models should be trained on user-generated data. Instructions for training the former are available on the CellProfiler documentation website, and we have included example pipelines for selecting worms and training the model in the GitHub repository.

For transgenic worms with fluorescent markers, we also chose to filter during post-processing rather than implementing a filter in the CellProfiler pipeline. This allows for labeling each worm as +/- in the final tidied data, providing a convenient within-well control population (transgenic strains generated with extrachromosomal arrays contain a mix of transgene^+^ and transgene^-^ worms). We trained a simple random forest on annotated worms that were labeled with *unc-122p*::*GFP*, which is fluorescent in only a handful of cells (Fig 5B). This classifier achieved 100% accuracy, and the most important variable in the model was the standard deviation of the fluorescence intensity (Fig 5C). Internally, we also use this model for classifying pharynx-labeled (*myo-2p*::*GFP*) transgenic worms.

Finally, we include a CellProfiler pipeline for the measurement of staining by a viability dye (CellTox), which we have used with both microfilariae and adult *C*. *elegans*, and simple modules for calculating total worm area, which we have optimized for assessing worm *in vitro* fecundity (Fig 4). We have previously shown that *in vitro* fecundity of *Brugia* spp. adults can be an important phenotypic measure for macrofilaricidal screens, and wrmXpress operationalizes the analytical side of this experimental protocol [16]. For the flatworm *S*. *mansoni*, fecundity measurement has historically been performed by hand [31–33], and to our knowledge this the first instance of an automated protocol for counting schistosome eggs in the same well in which the adult worms were cultured, now allowing for high-throughput assessment of schistosome fecundity. Indeed, we found that this phenotype is a more sensitive measure for praziquantel efficacy than *in vitro* measure of motility, with a racemic mixture having an IC50 80% lower than that of the active enantiomer IC_50_ for motility (14 nM vs 68 nM, Fig 4C) [34].

### wrmXpress is readily extensible

wrmXpress can be extended by developing new, isolated modules (e.g., Python scripts) that take the images from a single well, perform transformations/calculations on them, and output a single value. For instance, one can easily imagine a Python module that counts segmented objects in a well. The Python script can be written, added to the modules/ directory, added to the if/else loop in the wrapper script, and added as an option in the YAML configuration template. The module will be run independently, enabling safe, backwards-compatible engineering of new modules.

Likewise, new CellProfiler pipelines can also be easily implemented. In this case, a pipeline is developed in the CellProfiler GUI, exported as a .cppipe file, added to the cp_pipelines/pipelines/ directory, and added as an option in the YAML configuration template. As an additional step, a user must also add an R script that parses the input file names and generates the CSV file that is read by the LoadData module in CellProfiler.

wrmXpress does not have a GUI and therefore can only be extended by R and Python developers. However, we have taken great pains to make the addition of Python modules simple and barrier-free. Additionally, we have found that researchers without programming experience can develop pipelines using the CellProfiler GUI, which can then be integrated into the wrmXpress framework by novice developers. Lab specific documentation for extending wrmXpress can be found at http://www.zamanianlab.org/ZamanianLabDocs/pipelines_wrmxpress/, which may be instructive.

## Discussion

We view wrmXpress as a part of the next-generation of parasitic worm phenotyping toolkits, building upon important advances made by WormAssay/Worminator [3,4] and the WormToolbox [11] and enabled by high-content imaging. The software contains a variety of analytical modules that are optimized for experiments with worms, and new modules are being developed. For instance, we are actively experimenting with modules to count distinct classes of progeny (for instance, high-throughput *Brugia* spp. embryograms). High-throughput assessment of worm fecundity could make use of advanced culture media that enable *in vitro* reproduction [33].

Future developments in high-content phenotyping of worms likely include the utilization of deep learning frameworks for a variety of phenotypic endpoints. We have observed that drug treatment of worms can cause diverse, often ephemeral, motility behaviors that can be identified by eye, but as of yet cannot be classified computationally [10]. We have additionally observed that drug-induced worm death can result in one of a number of different worm postures, which we believe is related to drug mechanisms of action (MoA), in the same way that drug MoA can be parsed by classifying behavioral fingerprints in *C*. *elegans* [35]. Deep learning is well suited for each of these tasks, and the structure of wrmXpress is such that deep learning modules can easily be added. Indeed, these extensions are actively being developed.

Due to limitations in running CellProfiler in headless mode, wrmXpress cannot currently be run in parallel (i.e., analyzing individual wells by separate processors). However, high-throughput screens often generate dozens of plates per day, and wrmXpress is readily capable of analyzing plates in parallel by submitting separate jobs for each plate (or running separate commands on a local machine). Indeed, this is our current implementation with HTCondor [20]. However, future developments of wrmXpress could allow for well-based parallelization, either by changes to the handling and organization of input data, or by making use of Python’s multiple libraries for parallelization. Regardless, in our hands the analysis of a full 96-well plate routinely takes less than 2 hours using relatively modest hardware specifications (4 CPUs, 20 GB RAM).

wrmXpress will work out-of-the-box for all datasets generated with an ImageXpress (Molecular Devices), which is a popular platform for worm labs [13,15,36,37], and the whole-plate motility module can be implemented with single AVI files. For other endpoints, the image data must be structured as in Fig 1. However, the design of the pipelines is such that adding support for other platforms will be straightforward.

wrmXpress v1.3.0 can be downloaded from its public GitHub repository (https://github.com/zamanianlab/wrmXpress), and the Docker container that includes all dependencies is also available (https://hub.docker.com/r/zamanianlab/chtc-wrmxpress). Documentation can be found at the GitHub repository, and additional developer information can be found at http://www.zamanianlab.org/ZamanianLabDocs/pipelines_wrmxpress/.

## Supporting information

S1 File Building and evaluating models for classifying straightened worm objects. (PDF)

S1 Fig Evaluating trained Cellpose models. (PDF)

S2 Fig Correlations between diagnostic “flow clouds” and motility measurements. (TIF)
