# Supplementary figures and images for: wrmXpress: A modular package for high-throughput image analysis of parasitic and free-living worms
Source: PLoS Negl Trop Dis. 2022 Nov 18;16(11):e0010937. doi: 10.1371/journal.pntd.0010937 (PMC9718391; doi:10.1371/journal.pntd.0010937)

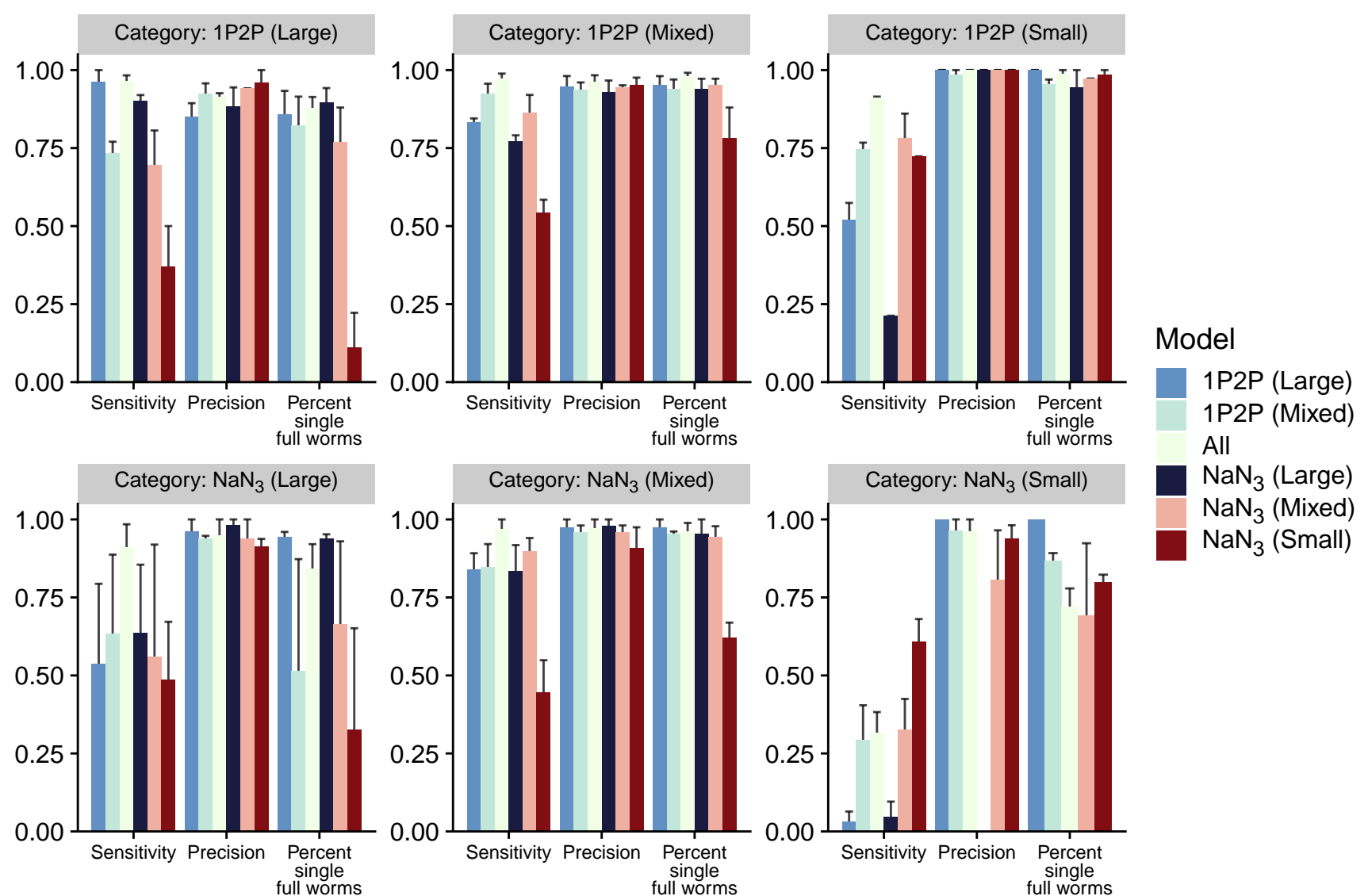

Supplement: S1 Fig — (PDF) [file pntd.0010937.s002.pdf]

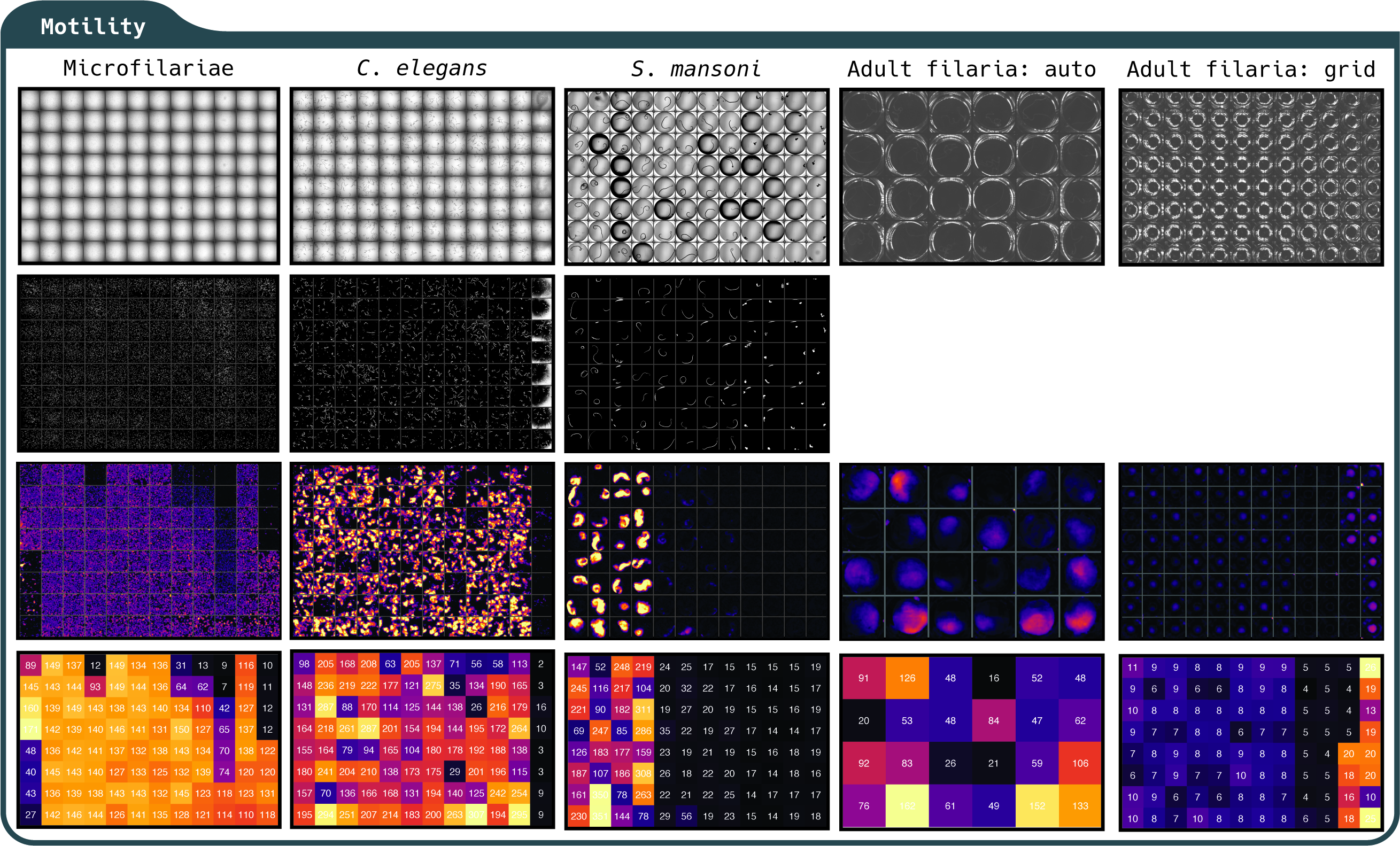

Supplement: S2 Fig — (TIF) [file pntd.0010937.s003.tif]
